# Nutrient Constituents, Bioactive Phytochemicals, and Antioxidant Properties of Service Tree (*Sorbus domestica* L.) Fruits

**DOI:** 10.3390/plants11141832

**Published:** 2022-07-13

**Authors:** Manol Ognyanov, Petko Denev, Nadezhda Petkova, Zhana Petkova, Magdalena Stoyanova, Peter Zhelev, Georgi Matev, Desislava Teneva, Yordan Georgiev

**Affiliations:** 1 Laboratory of Biologically Active Substances, Institute of Organic Chemistry with Centre of Phytochemistry, Bulgarian Academy of Sciences, 139 Ruski Blvd., 4000 Plovdiv, Bulgaria; petko.denev@orgchm.bas.bg (P.D.); desislava.teneva@orgchm.bas.bg (D.T.); yordan.georgiev@orgchm.bas.bg (Y.G.); 2 Department of Organic Chemistry and Inorganic Chemistry, Technological Faculty, University of Food Technologies, 26 Maritza Blvd., 4002 Plovdiv, Bulgaria; petkovanadejda@abv.bg; 3 Department of Chemical Technology, University of Plovdiv ‘Paisii Hilendarski’, 24 Tzar Asen Str., 4000 Plovdiv, Bulgaria; zhanapetkova@uni-plovdiv.net; 4 Department of Analytical Chemistry and Physicochemistry, Technological Faculty, University of Food Technologies, 26 Maritza Blvd., 4002 Plovdiv, Bulgaria; magdalena.stoianova@abv.bg; 5 Dendrology Department, University of Forestry, 10 Kliment Ohridski Blvd., 1756 Sofia, Bulgaria; petar.zhelev@ltu.bg; 6 Environmental Executive Agency (ExEA), Regional Laboratory Plovdiv, 1 Perushtitsa Str., 4004 Plovdiv, Bulgaria; georgi_matev@hotmail.com

**Keywords:** carbohydrates, protein, lipids, fatty acids, minerals, polyphenols, antioxidant activity

## Abstract

The current study aimed to determine the major and minor nutritional constituents of *Sorbus domestica* L. fruits. It was revealed that palmitic acid was the most commonly occurring saturated fatty acid, while linoleic acid represented the major polyunsaturated fatty acid. The sterol fraction consisted mainly of *β*-sitosterol. Small amounts of lipophilic pigments were quantified. Potassium, iron, and boron were the most abundant macro-, micro-, and ultra-trace elements. The amino acid composition analysis suggested that the non-essential amino acids predominated over the essential ones. Soluble sugars (fructose and glucose) represented a large part of the total carbohydrate content, but pectin formed the major part of polysaccharides. Malic acid was the most abundant organic acid whereas quercetin-3-*β*-glucoside, neochlorogenic, and 3,4-dihydroxybenzoic acids were the major phenolic constituents. Fruits exhibited free-radical scavenging and protecting ability against peroxyl and hydroxyl radicals. Service tree fruits provided valuable bioactive constituents having a high nutritional value and potential health benefits.

## 1. Introduction

Service tree (*Sorbus domestica* L.) is a deciduous and long-lived tree with small pear-shaped fruits (average size of 3 cm) that belongs to the Rosaceae family [1,2]. It has a relatively wide distribution across temperate central and southern Europe (the Balkans, Italy, and southern France), but it is also rarely distributed in the northern parts of Africa and western Asia [1,2,3]. Nevertheless, the service tree is considered an endangered species in some countries due its small proportion of domestication [2,4]. In Bulgaria, the service tree is known as ‘skorusha’ or ‘oskrusha’, and its (over)ripe fruits are often freshly consumed or processed into compôte, jams, jelly, or marmalade [5]. In traditional folk medicine, many people try to cure themselves of gastrointestinal (diarrhea, diabetes, vomiting) and urogenital (kidney stones) illnesses using the striped bark, young twigs, and fruits [1,5]. In general, the service tree is known for its beneficial properties, but nonetheless, it is usually only used by local people who live close to its habitats.

A thorough review of current scientific knowledge of the service tree revealed that scientists have conducted investigations, mainly into different ecological, morphological, and genetic aspects [1,2,3,4,5]. Unfortunately, however, few studies are focused upon the chemical composition and nutritional value of the service tree fruits [4,6,7,8,9,10,11,12]. A very minor part of the reports draws attention to the presence and accumulation of some minerals [6,13]. The other reports note evidence that fruits and their extracts are a rich source of phenolic compounds, and they possess a wide range of benefits including anti-inflammatory, antidiabetic, antimicrobial, and antioxidant [7,8,10,14]. Furthermore, in a series of articles, a team of scientists has reached a deep understanding of the phenolic composition of fruits at different maturity stages [9,11,12]. Very few scientists turn their attention to other plant parts such as leaves and their chemical constituents [15,16].

The main conclusion to be drawn from the abovementioned studies is that the main active constituents of the fruits and leaves of the service tree are different polyphenols (flavonoids, proanthocyanidins, etc.) mostly having antioxidant activity. However, the fact remains that details of the other phytochemical constituents of fruits are still scarce or completely missing in the available literature. Service tree fruits have not been exhaustively studied for their lipid constituents (fatty acids, phytosterols, and phospholipids). Moreover, there have not yet been any quantitative data about the presence of polysaccharides (cellulose, starch, and pectin), although they are very important active components responsible for the biological and functional properties of service tree fruits. In addition, there are very little data available about mineral composition, and what is more, the content of elements such as P, Se, B, Al, Co, As, Cd, Hg, Pb, etc., have not yet been evaluated. We could not find any detailed report about the presence and composition of organic and amino acids.

Therefore, the current study is chiefly concerned with conducting an investigation into the presence and composition of different major and minor phytochemical constituents, including lipids, proteins, carbohydrates, organic acids, amino acids, minerals, and polyphenols of service tree fruits collected in Bulgaria.

## 2. Results and Discussion

### 2.1. Protein Content and Amino Acid Composition

The protein content and results of the quantitative estimation of various amino acids are presented in Table 1. It can be seen that a small amount of protein was present in fruits (3.5% dw, 1.1% fw, 35 mg/g dw). It is not surprising, therefore, that 100 g of fresh service tree fruits provides a very small proportion (≤3.0%) of the intake of the protein requirement of 0.65 g/kg/day (for 60 kg bw). Furthermore, amino acid composition analysis revealed that the non-essential amino acids (55% of total) predominated slightly higher in comparison to the essential ones (45% of total). The non-essential glutamic and aspartic acids contributed to a large part of the total amino acid content (23%) like in most fruits. This suggested that some protein constituents were acidic in character. These negatively charged amino acids play a major role in proteins. The metal-binding sites of many proteins contain one or several aspartate and glutamate-side chains [17]. However, it should be noted that asparagine and glutamine, if present, may be deaminated to aspartic acid and glutamic acid during acid hydrolysis, and thus the determination of these constituents seemed to be in doubt. The acid hydrolysis used to liberate the acids also provided an adequate explanation for the lack of tryptophan and cysteine which may be recovered in a non-quantifiable yield or destroyed by oxidation under these conditions. Phenylalanine was found in smaller amounts among other amino acids. Together with threonine, they occupied not more than 13% of all essential amino acids. Glycine was also a minor constituent that represented nearly 10% of the total non-essential amino acid content. One further analysis of the data showed that amino acids with non-polar (hydrophobic) and uncharged side chains such as glycine, proline, alanine, valine, leucine, isoleucine, phenylalanine, and methionine were very important major contributors to the total amino acid content (45%) of the service tree fruits. Amino acids with uncharged, polar side chains (serine, threonine, tyrosine, asparagine, and glutamine), on the other hand, seemed to be minor contributors to the total content (9.2%). Amino acids carrying positively charged side chains (histidine, lysine, and arginine) comprised about 17% of the amino acid pool, and together with negatively charged Asp and Glu, made up about 40% of the total ones.

It is a well-known fact that a comparison between the amino acid content of investigated food and amino acid requirements is used for assessing the quality of protein in foods (in vitro). Accordingly, we were interested in calculating the amino acid score of fruits that reflected their overall nutritive value. Not surprisingly, by our calculations (Table S1), it became clear that the protein of the service tree fruits bore no comparison with that of bovine milk, although it seemed that it could contribute to the positive balance of His (167%) and Met (141%) [17]. Nevertheless, in comparison with the amino acid composition of ‘ideal’ protein, the service tree fruits had superior amounts of essential amino acids (His, Met, Val, Phe). Leucine and lysine were considered to be limited acids, not to mention the question of fruit protein digestibility (Table S1).

It is interesting to note that a lower ‘true’ value of the protein content (2.6% or 74.1% recovery of crude protein) of the service tree fruits was calculated when we summed the individual anhydrous amino acid values, assuming that there were not any free amino acids and that all of them were present in polymer form. However, it is important to remember that some amino acids (Trp, Cys, Asn, Gln) were recovered in low non-quantifiable yields, and thus the ‘true’ value would seem to be higher. Another interesting finding of the study was that 78.4% of total nitrogen was recovered in the form of amino acids—in other words, not all nitrogen could be ascribed to the protein. Therefore, about 22% of total nitrogen may be non-protein nitrogen (nucleotides, chlorophylls, polyamines, and amino alcohols of phospholipids, etc.).

There has not yet been any report, to our knowledge, that examined in detail the amino acid composition of service tree fruits, hence to compare with that reported in the current study. Incidentally, several points of similarity between the service tree and apple may provide a basis for comparison. Methionine, tyrosine, arginine, and proline, for example, predominated in the service tree fruits (Table 1), whereas apple offered superior amounts of aspartic and glutamic acids (1300 and 700 mg/g N) [18].

### 2.2. Lipid Composition

The results of the lipid composition analysis are summarized in Table 2. The crude lipid fraction represented 0.82% (*w*/*w*) of the dried fruits. It was evident that 16 fatty acids were identified and quantified, including 9 saturated and 7 unsaturated ones. Palmitic acid (16:0; 19.5%) was the most widely occurring saturated fatty acid followed by stearic acid (18:0; 3.3%), which was much less common. Both represented 93% of the total amount of saturated acids. Other saturated acids of chain length smaller than 16 carbon atoms (C_8:0_-C_14:0_) were present at very low levels (0.1–0.4%), in addition to a very small percentage of odd-chain fatty acids (15:0, 17:0), and arachidic acid. Therefore, those components were of lesser nutritional importance. Furthermore, it could be observed that unsaturated fatty acids predominated significantly higher in glyceride oil (75.5%) than in saturated fatty acids (24.5%). Linoleic acid (18:2) represented the major polyunsaturated fatty acid constituent (47.0%). Together with the monounsaturated oleic acid (18:1)—27.3%, they occupied nearly 98% of all unsaturated fatty acids detected in the lipid fraction. Linoleic and linolenic acids are essential fatty acids, and they must be additionally supplied by other sources.

Further useful information could be received from the fatty acid composition data such as PUFA/SFA ratio, AI, TI, and CI (Table S2). These values provide us with a view of the nutritional value and potential health benefits of the lipid fraction. What is more, these values also allow us to compare the results of our study with those of other studies. Keeping in mind a high PUFA content (48%), the value of the PUFA/SFA ratio was computed at 2.0, suggesting that the high intake of fruits would help reduce the risk of cardiovascular disease and oxidative stress. Ideally, it is recommended that the ratio should range in value from 1.0 to 1.5. From the lower values of AI (0.3) and TI (0.6) and the higher CI (3.8) value, it was also evident that the anti-atherogenic, anti-thrombogenic, and hypocholesterolemic fatty acids made up a large proportion of the total fatty acids. This suggested a high nutritional quality of the lipid fraction that might contribute to the minimization of a deficiency in essential fatty acids and consequently to the reduction of atherogenic and thrombogenic factors. It is worth mentioning that the fatty acid composition and corresponding AI and TI were nearly identical to those of soybean, corn, and sunflower oils. However, it stood in clear contrast to those of palm and coconut fats and olive oil [19].

Furthermore, we focused our attention on the quantification of sterol and tocopherol constituents (Table 2), which has not been carried out before. As can be seen, the sterol fraction consisted mainly of *β*-sitosterol (90%) which is also a major constituent of soybean and sunflower oils [17]. The latter sterol was accompanied by lower levels of stigmasterol (3.6%) and Δ^7^-stigmasterol (2.3%). Other minor sterol components were cholesterol (1.4%) and brassicasterol (1.1%). Sterols, together with phospholipids (3.6%), play a leading role in the lowering of blood cholesterol levels and as constituents of all biological membranes. Regarding tocopherols, there was only a small amount of them (27 mg/kg). Impressively, *α*-tocopherol was a key contributor (100%) to the total tocopherol content. This is not a surprise taking into consideration that the fruits not only have a low concentration of tocopherols but also have barely detectable levels of tocopherols other than *α*-tocopherol in contrast to leafy green vegetables. Nevertheless, daily intake of service tree fruits could serve as a beneficial supplement of *α*-tocopherol, which is an essential vitamin and antioxidant [17].

Lipophilic pigments (chlorophylls and carotenoids) were found in small quantities. Interestingly, among the chlorophylls, chlorophyll *b*, which is mainly responsible for the yellow-coloring of the fruits, was more predominantly present (24.2 mg/kg) than the blue-green chlorophyll *a* (*a*/*b* ratio = 0.56). This could be associated with the decomposition of chlorophyll *a* during fruit ripening and/or with specific environmental conditions, especially with low exposure to sunlight. The total chlorophyll content was comparable to those of commercial, red-skinned apple cultivars, and the carotenoid content was in agreement with previously reported values for apples, peas, and lemons (0.9–7.0 ppm) [17]. The vital role of carotenoids in achieving and maintaining health due to their provitamin and antioxidant activity should be emphasized. Chlorophylls, on the other hand, are often used as a marker of food quality.

### 2.3. Carbohydrate and Organic Acid Composition

Table 3 summarizes the carbohydrate compositional data of service tree fruits. The results indicated that carbohydrates made up the major fraction (about 45%) of service tree fruits’ dry matter (or nearly 14% fw). Interestingly enough, soluble sugars represented a large part of the total content (nearly 74%). Fructose (5.7% fw) was the main sugar found in the fruits, accounting for almost 56% of the total soluble sugars, followed by glucose (4.0% fw) and nonreducing sucrose (<0.5% fw). Fructose and glucose content was in complete agreement with an earlier study carried out by Brindza et al. who found reduced sugar content not more than 14–16% fw in fruit pulp of *S. domestica* [4]. Sorbitol, a member of sugar alcohols mainly having an osmotic regulation function, was quantified in a small amount.

The organoleptic characteristics of fruits could be obtained by calculating sucrose-glucose and glucose-fructose ratios, respectively (Table S3). It was evident that the major contributor to the sweetness index (56.89) was fructose (Glc/Fru = 0.72). The sour index was calculated to be 1.47, which was near that of beverages prepared from apple fruits. The maturity index, an important indicator of commercial and sensory ripeness of fruits, was estimated to be 21.80, which was comparable to reported values for peach and apple cultivars [20]. A higher value of the maturity index was fairly typical of sour–sweet to sweet fruits (17–24) and it suggested a good nutritional and organoleptic quality.

Furthermore, service tree fruits were investigated for their polysaccharide constituents. As shown (Table 3), the uronic acids, which adequately reflected the amount of an acidic polysaccharide such as pectin, were predominantly present in the fruit (3.1% dw, ~1.0% fw). This suggested that pectin formed the major component of polysaccharides (nearly 48%) in service tree fruits. Pectin, a water-soluble dietary fiber, is used in the food industry due to its gelling properties. What is more, increased consumption of pectin is linked to many positive health benefits, especially immunomodulation in the digestive tract [21]. Cellulose (0.7% fw) was observed to be the second most abundant polysaccharide constituent (35%), whereas starch, whose content significantly decreases in the ripened fruits, constituted the minor part of the total carbohydrate content. Thus, service tree fruits could be evaluated as a good source of available carbohydrates and dietary fibers. The intake of 100 g of fresh fruits will provide about 11% of the daily requirements of total carbohydrates (130 g/d). It can also cover about 8% (2 g/100 g fw) of the recommended amounts of dietary fiber (25 g/d). Since carbohydrates represented a major part of dry matter constituents, it is worth estimating their caloric value, just bearing in mind the energy conversion factor for soluble sugars (3.75–4.0 kcal/g) and commonly eaten foods that contain a mixture of fermentable and nonfermentable fibers (0–1.9 kcal/g). It was calculated that carbohydrates in a serving of fruit (100 g fw) will provide the consumer with nearly 45 kcal as a greater share of energy came from sugars (90%), while dietary fiber covered only a minor part (10%). As regards the last point, it should be mentioned that carbohydrates covered not more than 2.5% of the average daily energy requirement of 2000 kcal.

Furthermore, we focused our attention on the investigation of organic acid constituents (see Table 3) because organic acids have a closely related metabolism to carbohydrates. As can be seen, malic acid, one of the Krebs cycle acids, was the most abundant (360 mg/100 g fw), representing 74% of total organic acids. Malic acid content appeared to be quite comparable to the values reported for peach, pear, and strawberry, but it was four times lower than in apples (14.7 mg/g fw). It is widely considered that malic acid makes an important contribution toward providing the substrate used for gluconeogenesis, energy utilization, and pH regulation in fruits for a short period [22]. In addition, service tree fruits contain quinic acid in an appreciable amount (112.7 mg/100 g fw). By comparison, apples were characterized by a lower amount of quinic acid (0.2–0.7 mg/g fw), whereas medlar, peach, and blackcurrant showed similar levels (1–2 mg/g fw) [22]. In most fruits, quinic acid plays a key role as a reserve compound in phenolic biosynthesis, especially chlorogenic acid. It was easy to identify tartaric acid as a minor constituent in fruits, whereas ascorbic, shikimic, citric, and α-ketoglutaric acids were qualitatively detected, but these acids appeared to be present in non-quantifiable levels (Table 3). Nonetheless, one thing was for sure—their quantities did not exceed those in banana, pear, blueberry, and raspberry, for example [22].

### 2.4. Mineral Composition

Table 4 provides data for the total ash content and mineral composition of service tree fruits. As can be seen, potassium (4986 mg/kg; 155 mg/100 g fw) and phosphorus (400 mg/kg; 12.4 mg/100 g fw) were the most abundant macrominerals. The level of potassium, however, bore no comparison with that in well-known sources such as apricots, rose hips, and blackcurrant, but it was comparable to that found in apple, strawberry, or sour cherries [17]. A previous study by Majić et al. showed that service tree fruits collected in Croatia were also rich in potassium [6]. Together, potassium and phosphorus accounted for 55% of the total ash content (nearly 1% *w*/*w*) or more than 98% of the total amounts of macroelements detected. There is a complex interaction between macrominerals which are to a large extent actively involved in the regulation of acid–alkali balance. Potassium, for instance, is mainly localized within the cells and affects the osmotic pressure in the tissues. Moreover, potassium and sodium have a significant role to play in maintaining electrochemical gradients and secondary active transport in living cells. As a consequence, it exerts a diuretic effect, which can effectively be evaluated by the magnitude of the potassium-to-sodium ratio. The service tree fruits were characterized by a very high K/Na ratio (498:1) comparable to those of other medicinal plants (chamomile, linden, chicory) [23]. Therefore, this fact would seem to indicate that fruits are more likely to affect the sodium-induced hypertensive effect. Despite this, it was calculated that a rather low level of the recommended daily intake of potassium (25%/100 g dw or 7.7%/100 g fw) can come from these fruits, and thus the latter statement is open to doubt. This value was calculated by using 2.0 g as a recommended daily intake of K and assuming that 100% of the ingested element is absorbed [24]. Nevertheless, the consumption of service tree fruits could contribute to a positive K balance. Regarding other macroelements, magnesium, calcium, and sodium were found in smaller amounts (Table 4). Together they represented only 1.5% of the total macroelements; therefore, it seemed unreasonable to expect the service tree fruits to supply a considerable proportion of the intake of these macroelements (<1%/100 g fw). They are closely involved in the activation of many enzymes associated with energy conversion, in addition to the abovementioned functions.

Furthermore, a total of nine essential microelements were quantified. Iron was the most abundant (40.6 mg/kg), followed by copper and zinc (2.2 and 1.3 mg/kg, respectively). Iron, which is an essential constituent of a large number of enzymes, hemoglobin, and myoglobin pigments, accounted for almost 90% of the total, while the remaining part included the other microminerals (Table 4). The iron content (1.3 mg/100 g fw) in the service tree fruits was distinctly superior in comparison to that of fruits such as apples, oranges, apricots, and plums [17]. A daily intake of 100 g of fresh fruits can cover nearly 16% of the requirement (8 mg/day) of iron if its absorption is not disturbed. However, the value sounded very far-fetched, keeping in mind the low extent of iron absorption typical of vegetables and fruits [24]. Service tree fruits provided a relatively small proportion of the daily intake (900 µg) of copper (nearly 8%/100 g fw). It is an essential element responsible not only for iron uptake by the organism but also for the activity of enzymes such as polyphenol and ascorbate oxidase which cause fruits to brown. It is worth paying attention to chromium—an essential trace element that functions as an insulin and glucose level regulator. Inasmuch as 100 g of fresh fruits was estimated to contain 3.7 µg of chromium, a person who had consumed this amount of fruit per day covers nearly 11% of the recommended intake (35 µg).

Table 4 contains an important piece of information relating to the levels of ultra-trace elements and toxic metals found in service tree fruits. It is extremely debatable whether aluminum, arsenic, and boron have any benefit to human health, but the very negative effect of the remaining elements (Cd, Hg, and Pb) is a well-known fact. Aluminium and boron accounted for 50% and 21% of the total amount of the elements in this group. One of the most interesting things was that the consumption of 100 g of fresh service tree fruits may meet about 17% of the requirement of boron (1 mg/day). In other words, one serving of fruits provided the highest intake of boron among all other minerals. Boron is a constituent of rhamnogalacturonan-II fragment that composes pectin in particular, and it seems that it plays an important part in the metabolism (Ca, Mg, vit. D) and health of the bones. The boron content (167 µg/100 g fw) was comparable to that in other fruits (apples, oranges, peaches, grapes): 107–187 µg/100 g fw. Further and most importantly, the concentration of toxic elements was well below the permitted levels. Service tree fruits (100 g fw) contained only very small quantities of Cd (1.5 µg), Pb (0.6 µg), and Hg (0.9 µg). For this reason, the risk of exceeding the provisional tolerable daily intake limit of these elements (Cd—60 µg; Pb—214 µg; Hg—43 µg), suggested for 60 kg bw by the Joint FAO/WHO Expert Committee, was considerably reduced even consuming more than one serving per day [25]. The value of Cd was higher than in a previous study, while that of Pb was significantly lower [13].

### 2.5. Phenolic Compounds and Antioxidant Activities

The content of total polyphenols, flavonoids, and tannins was investigated, and the results obtained together with quantitative compositional data are presented in Table 5. Total phenolic content (~36 mg/g dw; 1109 mg/100 g fw) was quite comparable to that reported for mesocarp and exocarp of service tree fruits collected in Croatia [6]. By comparison, it was significantly higher than in most widely consumed fruits [26]. Regarding total flavonoids, however, some discrepancies in the values existed. Results also showed that quercetin-3-*β*-glucoside was found in the highest amount, while the other flavonoids appeared to be minor constituents in service tree fruits. This result confirmed the findings of previous studies that 3-*O*-glycosylated quercetin was more prevalent among other flavonoids [7,12]. Furthermore, it can be seen that neochlorogenic and 3,4-dihydroxybenzoic acids were major constituents (97%) among phenolic acids detected, whereas ellagic and ferulic acids were found in small amounts. The presence of different phenolic constituents such as hydroxybenzoic acid derivatives in service tree fruits has already been reported [12]. However, in contrast to the study conducted by Piagnani et al., gallic acid was not detected in the current study [8]. The most likely explanation for the discrepancy in phenolic composition could be fruit material origin, its maturity, environmental factors that affect fruit growing, and, last but not least, experimental conditions.

In general, phenolic constituents contribute not only to the nutritional properties (taste, flavor, color) of foods but also serve a useful function as antioxidants, inhibitors of lipid peroxidation, and enzymatic browning of fruits and vegetables. Thus, we were actively interested in determining the antioxidant activity of service tree fruits (Table 5). The results indicated that fruits had a better ability to scavenge DPPH-radical than the ability to reduce ferric ions. In previous studies, it has been demonstrated that different extracts of *S. domestica* exhibited antioxidant activity evaluated by DPPH, FRAP, and ABTS [6,7,9]. However, we could not draw comparisons with the results, because these authors not only used different extracts/parts for testing the antioxidant activity but also different units of measurement. In addition, the current study also employed ORAC and HORAC methods for a more exhaustive assessment of antioxidant activity. The ORAC method evaluates the peroxyl radical chain-breaking ability of antioxidants by the hydrogen atom transfer pathway, while the HORAC measures metal-chelating radical prevention activity of the sample. The results suggested that service tree fruits’ constituents had much higher free-radical scavenging ability against the peroxyl radical rather than the protection ability against the formation of the hydroxyl radical (the ORAC–HORAC ratio stood at 10:1). Moreover, it was particularly interesting to compare our results with a previously published study on Bulgarian fruits [26]. It came as a genuine surprise to find that service tree fruits had a powerful ORAC antioxidant activity (about 200 µM TE/g fw) comparable only to those of rosehip and elderberry. On the other hand, it far exceeded those of apple, apricot, peach, and even that of chokeberry (161 µM TE/g fw), well known for its high antioxidant activity [26,27].

## 3. Materials and Methods

### 3.1. Plant Material

The plant material was picked in the surroundings of Kosti village (Tsarevo Municipality, Burgas Province, 42°03′ N, 27°46′ E) and immediately transported to the laboratory. We employed 30 fruits, a quantity high enough for morphometric characterization. They were randomly selected from a higher number of fruits which was collected from three trees located relatively close to each other. Fruits were separated from leaves and small twigs, then gently washed clean without damaging the skins and cut into half or quarters. Furthermore, they were freeze-dried, milled into flour, and stored in an exicator, in a Ziploc bag at room temperature. The average (*n* = 30) mass and length of the fresh fruits were 12.5 ± 2.9 g and 2.6 ± 0.3 cm, respectively. The dry matter content of fruits was 30.0 ± 0.2%; 100 g of dry fruits corresponded to 322 g fresh ones—an amount equivalent to 9–10 fresh fruits. The fruit material was identified by the references of the Herbarium of the Institute of Biodiversity and Ecosystem Research where a voucher specimen (SOM 177 441) was deposited.

### 3.2. Proximate Composition Analysis

For the determination of moisture content, the milled sample (~1.5 g) was dried in an automated moisture analyzer (KERN DLB, Germany) at 105 °C until constant weight. Ash content was determined as the pulverized sample (0.5 g) was placed in a crucible and ignited in a muffle furnace at 550 °C until there was no change in the mass of the sample. For the estimation of crude lipid content, the ground sample (10.0 g) was packed in a cellulose thimble and subjected to an exhaustive extraction with *n*-hexane (500 mL) for 8 h in a Soxhlet extractor. The obtained crude extract was dried under vacuum, and its weight was used for the calculation of the lipid content. The crude protein content was evaluated by the micro-Kjeldahl method (N × 6.25). The determination of nitrogen expressed as ammonia content of the digested sample was performed by the acetylacetone–formaldehyde colorimetric method using ammonium sulfate as a standard [28]. The total carbohydrate content of the fruits was analyzed by the phenol–sulfuric acid method using a mixture of glucose and galacturonic acid (1.5:1) for the calibration curve construction [29]. The dried sample was solubilized in 72% (*w*/*w*) H_2_SO_4_ (1 h, 30 °C), and after dilution with water to 1 M H_2_SO_4_, hydrolysis was completed in 3 h at 100 °C. The obtained hydrolyzate was used as a sample for analysis. The absorbance was measured at 490 nm.

### 3.3. Amino Acid Composition

For the estimation of amino acid composition, the sample (300 mg) was hydrolyzed (5 mL, 6N HCl) in a sealed glass ampule at 105 °C for 24 h. The sample was vacuum-dried, reconstituted in 10 mL 20 mM HCl, and filtered. A total of 20 microliters of the collected filtrate was derivatized using an AccQ•Fluor kit (WATO52880, Waters Corp., Milford, NH, USA) according to the instruction manual of the manufacturer. The resulting derivatives were separated on an ELITE LaChrom HPLC system (VWR^™^ Hitachi, Tokyo, Japan), equipped with a diode array detector and a reversed-phase column C18 AccQ•Tag (3.9 mm × 150 mm) operating at 37 °C. The volume of the injected sample was 20 µL. The elution was performed with a flow rate of 1.0 mL/min with two mobile phases: (A) WATO52890 buffer (Waters Corp., Milford, NH, USA) and (B) 60% acetonitrile. The gradient mode was set as follow: 0–0.5 min 100–98% A, 0.5–15 min 98–93% A, 15–19 min 93–90% A, 19–32 min 90–67% A, 34–37 min 0% A, and 38–64 min 100% A. The different amino acid derivatives were detected at 254 nm.

### 3.4. Fatty Acid and Sterol Composition

The fatty acid composition was determined by gas chromatography (GC) after transmethylation of the sample (2% H_2_SO_4_ in CH_3_OH at 50 °C). Fatty acid methyl esters (FAMEs) were purified by thin-layer chromatography (TLC) on 20 × 20 cm plates covered with a 0.2 mm silica gel 60 G (Merck) layer with mobile phase hexane:diethyl ether (97:3, *v*/*v*). GC was performed on a HP 5890 series II (Hewlett Packard GesmbH, Vienna, Austria) apparatus equipped with a 75 m × 0.18 mm (I.D.) × 25 μm (film thickness) capillary column Supelco and a flame ionization detector. The column temperature was programmed from 140 °C (5 min), at 4 °C/min to 240 °C (3 min); injector and detector temperatures were kept at 250 °C. Hydrogen was used as a carrier gas at a flow rate of 0.8 mL/min, and a split ratio of 1:50. The identification of fatty acids was performed by comparison of retention times with those of a standard mixture of FAME (Supelco, Bellefonte, PA, USA 37 comp. FAME mix) [30].

The quantification of sterols was carried out spectrophotometrically after their isolation from unsaponifiable material by TLC on a Silica gel 60 G plate. A mixture of diethyl ether and hexane (1:1 *v*/*v*) and methanol were employed as a developing solvent and spraying reagent, respectively. Unsaponifiables were obtained after saponification of the oil by boiling under reflux with an ethanolic 2 M KOH solution and extraction by *n*-hexane. The different sterols were identified on the same apparatus used for fatty acid composition analysis with a 25 m × 0.25 mm DB-5 capillary column and flame ionization detector. The temperature gradient was from 90 °C (hold 2 min) up to 290 °C at a rate of change of 15 °C/min and then up to 310 °C at a rate of 4 °C/min (hold 10 min); detector temperature—320 °C; injector temperature—300 °C and carrier gas—hydrogen. Identification was confirmed by the comparison of retention times with those of a standard mixture of sterols [30].

### 3.5. Phospholipid and Tocopherol Content

The quantification of phospholipids was carried out colorimetrically by measuring the phosphorous content at 720 nm by the sulfate–molybdate reagent after mineralization of the oil with a mixture of perchloric acid and sulphuric acid (1:1, *v*/*v*).

Tocopherols were determined directly in the oil by high-performance liquid chromatography using a Merck-Hitachi apparatus equipped with a 250 mm × 4 mm Nucleosil Si 50-5 column and a fluorescent detector Merck-Hitachi F 1000. The operating conditions were as follows: a mobile phase containing *n*-hexane:dioxan, 96:4 (*v*/*v*), a flow rate of 1.0 mL/min, excitation 295 nm, emission 330 nm. Twenty microliters of the sample (1% solution of crude oil) was injected. The tocopherols were identified and quantified by comparing the retention time and peak areas of the sample with those of the standard solutions [30].

### 3.6. Total Carotenoid and Chlorophyll Content

The total content of chlorophylls and carotenoids was determined using acetone as a solvent. Absorbance was measured at three different wavelengths 662, 644, and 470 nm according to Lichtenthaler and Wellburn [31].

### 3.7. Uronic Acid, Cellulose, and Starch Content

For the estimation of the uronic acid content of fruits, an automated 3-phenylphenol analysis was performed by a continuous flow analyzer Skalar San^++^ system (Skalar Analytical BV, Breda, The Netherlands) according to the instructions of the manufacturer. Absorption was measured at 530 nm, and galacturonic acid (12.5–100.0 μg/mL) was used for a calibration curve construction. Initially, the sample was given a preliminary threefold extraction with 70% (*v*/*v*) aqueous ethanol at 50 °C for 1 h to remove small molecules. The solids were separated by centrifugation (18.187× *g*) before each repetition. Furthermore, the residue was washed twice with acetone at room temperature and vacuum-dried. Finally, the sample was hydrolyzed as described above (Section 3.2), and an aliquot of hydrolysate was used as a sample for analysis.

The quantitative estimation of cellulose was performed gravimetrically. Briefly, a sample (0.5 g) was gently boiled (30 min) with 25 mL of acetic acid-HNO_3_ reagent (acetic acid:H_2_O:HNO_3_ 8:2:1 *v*/*v*/*v*) in a round-bottom flask fitted with a reflux condenser. After cooling, the insoluble residue was filtrated through a sintered glass filter (G3) under vacuum, washed with deionized water to neutral pH, then with ethanol (96% *v*/*v*), and finally with an excess of petroleum ether. The obtained residue was dried in a laboratory oven at 50 °C to a constant weight. The resulting cellulose was corrected for its ash content.

A combination of the *α*-amylase/amyloglucosidase method for conversion of starch into glucose and the colorimetric glucose oxidase/peroxidase/4-aminoantipyrine (GOPOD) method of measuring glucose content was employed to determine the total starch content. The analysis was conducted according to the analytical protocol described by Hall [32].

### 3.8. High-Performance Liquid Chromatography Analysis of Available Carbohydrates

In a plastic centrifuge tube, about five grams of chopped fresh fruits were weighed out. To this, distilled water (25 mL) was then added. The tube was placed in an ultrasonic bath (Siel UST 5.7-150, Gabrovo, Bulgaria), and an ultrasound-assisted extraction was performed with a frequency of 35 kHz (300 W) at 50 °C. After heating for 20 min, the tube was cooled in running water, then filtrated successively through a paper and PTFE filter (0.45 µm). The quantitative chromatographic separation of free sugars was carried out on a Shodex^®^ Sugar SP0810 (300 mm × 8.0 mm i.d.) column having Pb^2+^ as a counter ion and a Shodex SP-G guard column (5 μm, 6 mm × 50 mm) (Shodex Co., Tokyo, Japan) with ultra-purified water (Adrona B30, Riga, Latvia) as a mobile phase. A Shimadzu HPLC system, equipped with an LC-20 AD pump and a Shimadzu RID-10A detector was used. The volume of the sample was 20 μL, and it was eluted at 85 °C and a flow rate of 0.5 mL/min.

### 3.9. High-Performance Liquid Chromatography Analysis of Organic Acids

A total of 1 g of the ground sample was extracted with 30 mL of water for 1 h at 30 °C shaking on a thermostatic water bath (NÜVE, Turkey). The residue and extract were then separated through a Büchner funnel (filter paper, KA-4, Czechia) and additionally filtrated through a PTFE filter (0.45 µm). The sample was further passed through a Sep-Pak^®^ plus C18 RP cartridge (Waters Corp., Milford, NH, USA), and the eluate was taken for chromatographic analysis. The quantitation of organic acids was conducted on a Nexera-*i* LC2040C Plus UHPLC system (Shimadzu Corporation, Kyoto, Japan) with a UV detector. The system was controlled by LabSolutions (ver. 5.98) software (Shimadzu Corp.). The separation was performed on a Mediterranea Sea18 (5 µm, 4.6 mm × 150 mm; Teknokroma^®^, Spain) column at 25 °C and a flow rate of 1.0 mL/min. Then, 20 microliters of the sample was auto-injected and eluted isocratically using a 25 mM solution of K_2_HPO_4_ in water as a mobile phase, whose pH was finely adjusted to 2.4 with H_3_PO_4_. The UV detector was set at 210 nm. The concentration of each organic acid in the sample was calculated using a calibration curve obtained by using five different concentrations for each acid. The peak corresponding to different acids was confirmed by comparison of the retention time with that of the standards.

### 3.10. Mineral Composition

The mineralization of the freeze-dried fruits (0.5 g) was performed in a heat-controlled microwave system with 9 mL of HNO_3_ (Supra Pure Metal, 65%) in a closed vessel system. The digestion was carried out on Milestone ETHOS PLUS lab station with MPR-300/12S medium pressure rotor and heating in 2 stages (5 and 10 min at 180 °C up to 1000 W power, respectively). The micro and trace elements (Ni, Cu, Zn, As, Cd, Pb, Cr, Mn, Co, Hg, Al, Se, Ba, Sr) of the investigated sample were analyzed using ICP-MS Agilent 7500 (G3272B) (Agilent Technologies, Inc., Tokyo, Japan) spectrometer. The major elements (K, Ca, Na, Mg, Fe) were analyzed by AAS (PerkinElmer 3030 B, Waltham, MA, USA), while Mo, Ba, B, and P elements were quantified by ICP-OES (Prodigy 7, Teledyne Technologies Incorporated, Hudson, NY, USA). The results were expressed in mg/kg using calibration standards. The reference material was dried peach leaves of the Coronet variety (Standard Reference Material^®^ 1547, National Institute of Standards and Technology, Gaithersburg, MD, USA).

### 3.11. Total Phenolic, Flavonoid, and Condensed Tannin Content

The freeze-dried and ground fruits (0.5 g) were extracted with 40 mL of solvent containing 80% acetone in 0.5% formic acid at room temperature on a magnetic stirrer for 1 h. Then, the sample was centrifuged (6000× *g*, 20 min), and the supernatant was collected. The total phenolic content was determined according to the method of Singleton and Rossi with Folin–Ciocalteu’s reagent [33]. Gallic acid (10–200 μg/mL) was employed as a calibration standard.

About 1.0 g of the freeze-dried and ground sample was extracted with 40 mL of solvent containing 80% ethanol in 0.5% formic acid on a magnetic stirrer at room temperature for 1 h. The sample was centrifuged (6000× *g*) for 20 min, and the clear supernatant was used for total flavonoid content analysis. It was determined according to the method of Chang et al. with AlCl_3_ reagent [34]. The calibration curve was constructed with quercetin dihydrate (10–200 mg/L). Part of the extract was taken for the determination of tannins by methylcellulose precipitation assay using epicatechin aqueous solutions as a standard [35].

### 3.12. High-Performance Liquid Chromatography of Phenolic Components

The qualitative and quantitative detection of phenolic components was performed on an HPLC system Agilent 1220 (Agilent Technology, Santa Clara, CA, USA) fully equipped with a binary pump and a UV–Vis detector (Agilent Technology, USA). The separation was performed on an Agilent TC-C18 column (5 μm, 4.6 mm × 250 mm) at 25 °C and a wavelength of 280 nm with two mobile phases: (A) 0.5% acetic acid and (B) 100% acetonitrile with a flow rate of 0.8 mL/min. The gradient elution started with 14% (B), between 6 and 30 min, linearly increased to 25% (B), and then to 50% (B) at 40 min. The identification of compounds was confirmed by comparison of retention times utilizing standard solutions and standard calibration curves of different phenolics. As a sample for the phenolic profiling, an acidified acetone extract prepared as described in 3.11 was used. The corresponding calibration curves of the authentic standards used are presented as supplementary files (Figures S1 and S2).

### 3.13. In Vitro Antioxidant Activity Assays

Oxygen radical absorbance capacity (ORAC) and hydroxyl radical averting capacity (HORAC) were measured according to the methodology used by Denev et al. [27]. Both analyses were carried out on a FLUOstar OPTIMA plate reader (BMG Labtech, Ortenberg, Germany); 1, 1-Diphenyl-2-picrylhydrazyl (DPPH) radical-scavenging ability and ferric reducing antioxidant power (FRAP) assay and the preparation of extracts were carried out as described by Ivanov et al. [36]. As a sample for ORAC and HORAC analyses, an acidified acetone extract prepared as described in Section 3.11 was used, while for the estimation of DPPH and FRAP activities, a 70% ethanol extract was employed [36].

### 3.14. Lipid and Carbohydrate Indexes Calculation

A polyunsaturated-to-saturated fatty acids (PUFA/SFA) ratio, atherogenic (AI), thrombogenic (TI), and cholesterolemic indexes (CI) were calculated from data from the fatty acid composition following the formulas described in Petkova, Antova, and Angelova-Romova [30]. The maturity index and other carbohydrate indexes were calculated as described in Mihaylova et al. [20].

### 3.15. Statistics

The experimental data were subjected to an analysis of variance, at the confidence level of *p* = 0.05, using Statistica v. 8.0 software (Statsoft, Inc., Tulsa, OK, USA). A Fisher test was used for the determination of statistically significant differences if applicable.

## 4. Conclusions

For the first time, we reported an in-depth study that provided valuable insight into the phytochemical composition of service tree fruits. To our knowledge, the current study reveals for the first time the lipid (saturated and unsaturated fatty acids, phytosterols, and phospholipids) composition, the minerals (P, Se, B, Al, Co, As, Cd, Hg, Pb), the polysaccharides (cellulose, starch, and pectin), the amino acids, and the organic acid constituents of service tree fruits. Thus, we believe we not only fill the gaps in scientists’ knowledge, but also form a solid basis for a better assessment of the potential for practical applications. The results suggest that a regular intake of fresh fruits may contribute to making up a deficiency of important nutrients. It may contribute, to a higher extent, to maintaining a positive nutritional balance, especially of essential unsaturated fatty acids, potassium, iron, boron, histidine, methionine, and dietary fibers. Therefore, this unique combination of bioactive constituents may help reduce the risk of cardiovascular disease and oxidative stress. In addition, it seems reasonable to suggest that flour made from whole-dried service tree fruits would be a suitable form of practical application used in cooking for making dietary fiber-enriched bread and cakes. Moreover, it can be incorporated as an active natural ingredient in functional beverages and foods.

## Figures and Tables

**Table 1:** Crude protein content and amino acid composition of service tree fruits.

Crude Protein (N × 6.25), *w*/*w*%		3.5 ± 0.2	
A. Essential amino acids	mg/g sample	mg/g N	g/100 g protein
Valine (Val)	2.0 ± 0.3	357	5.8
Leucine (Leu)	1.9 ± 0.1	339	5.5
Isoleucine (Ile)	1.4 ± 0.2	250	3.9
Phenylalanine (Phe)	0.7 ± 0.08	125	2.1
Tryptophan (Trp)	-	-	-
Methionine (Met)	1.4 ± 0.1	250	4.1
Threonine (Thr)	1.1 ± 0.0	196	3.2
Histidine * (His)	1.6 ± 0.1	285	4.7
Lysine (Lys)	1.6 ± 0.1	285	4.6
Arginine ** (Arg)	2.1 ± 0.1	375	6.1
Total	13.8	2462	40
B. Nonessential amino acids			
Glycine (Gly)	1.6 ± 0.2	285	4.6
Alanine (Ala)	2.4 ± 0.2	428	6.8
Proline (Pro)	2.4 ± 0.1	428	7.0
Serine (Ser)	1.7 ± 0.1	303	4.7
Cysteine (Cys)	-	-	-
Tyrosine (Tyr)	1.7 ± 0.2	303	4.9
Asparagine (Asn)	-	-	-
Glutamine (Gln)	-	-	-
Aspartic acid (Asp)	4.0 ± 0.2	714	11.2
Glutamic acid (Glu)	3.0 ± 0.1	535	8.7
Total	16.8	2996	47.9

* essential for infants; ** “semi-essential” amino acid.

**Table 2:** Lipid composition of service tree fruits.

Total Lipids, *w*/*w*%		0.82 ± 0.07 ^a^
A. Saturated fatty acids, %		24.5
C8:0	Caprylic acid	0.4 ± 0.05 ^b^
C10:0	Capric acid	0.2 ± 0.01
C12:0	Lauric acid	0.1 ± 0.03
C14:0	Myristic acid	0.4 ± 0.03
C15:0	Pentadecylic acid	0.2 ± 0.03
C16:0	Palmitic acid	19.5 ± 0.6
C17:0	Margaric acid	0.3 ± 0.07
C18:0	Stearic acid	3.3 ± 0.3
C20:0	Arachidic acid	0.1 ± 0.02
B. Unsaturated fatty acids, %		75.5
C14:1	Myristoleic acid	0.1 ± 0.02
C16:1	Palmitoleic acid	0.1 ± 0.04
C18:1	Oleic acid	27.3 ± 0.3
C18:2	Linoleic acid	47.0 ± 0.2
C18:3	Linolenic acid	0.5 ± 0.2
C20:3	Eicosatrienoic acid (Dihomo-*γ*-linolenic acid)	0.2 ± 0.02
C20:4	Eicosatetraenoic acid (Arachidonic acid)	0.3 ± 0.04
C. Sterols, %		1.1 ± 0.1
	Cholesterol	1.4 ± 0.02 ^c^
	Brassicasterol	1.1 ± 0.04
	Stigmasterol	3.6 ± 0.1
	*β*-Sitosterol	90.2 ± 0.3
	Δ^5^-Avenasterol	0.6 ± 0.02
	Δ^7^-Stigmasterol	2.3 ± 0.1
	Δ^7^-Avenasterol	0.8 ± 0.1
D. Phospholipids, %		3.6 ± 0.1
E. Tocopherols, mg/kg		27.0 ± 2.0
F. Carotenoids, mg/kg		7.6 ± 0.5 ^d^
G. Total chlorophylls, mg/kg		37.8 ± 0.8 ^d^
	Chlorophyll *a*	13.7 ± 0.5
	Chlorophyll *b*	24.2 ± 0.3

^a^ Total lipids as *w*/*w*% of dried fruits, while lipid fractions are expressed as a percent of the total lipids: ^b^ % of total fatty acids; ^c^ % of total sterols; ^d^ mg/kg of dried fruits.

**Table 3:** Carbohydrates, organic acids, and total titratable acidity of service tree fruits.

A. Total Carbohydrate, *w*/*w*%	44.4 ± 1.7
Cellulose	2.3 ± 0.2
Starch	1.1 ± 0.1
Uronic acids (anhydrous)	3.1 ± 0.3
Glucose (Glc)	13.0 ± 1.3
Fructose (Fru)	18.2 ± 1.1
Sucrose (Suc)	1.5 ± 0.1
Sorbitol	1.6 ± 0.1
B. Organic acids, mg/100 g	
Malic acid	1160 ± 130
Quinic acid	363 ± 39
Ascorbic acid	<50 ^a^
Shikimic acid	<25 ^a^
Citric acid	<50 ^a^
α-Ketoglutaric acid	<50 ^a^
Tartaric acid	44 ± 5
C. Total titratable acidity (g malic acid/100 g)	1.5

^a^ under the limit of detection for this method.

**Table 4:** Total ash content and mineral composition of service tree fruits.

Total Ash, *w*/*w*%	0.98 ± 0.01
Macroelements	Content, mg/kg
Potassium (K)	4986.5 ± 85.5
Phosphorus (P)	400.0 ± 15.5
Magnesium (Mg)	40.9 ± 2.1
Calcium (Ca)	31.6 ± 1.8
Sodium (Na)	10.0 ± 1.0
Micro (Trace) Elements	
Iron (Fe)	40.6 ± 2.5
Copper (Cu)	2.2 ± 0.2
Zinc (Zn)	1.3 ± 0.2
Manganese (Mn)	0.9 ± 0.1
Chromium (Cr)	0.12 ± 0.02
Selenium (Se)	<0.1
Cobalt (Co)	0.07 ± 0.01
Molybdenum (Mo)	0.01 ± 0.00
Nickel (Ni)	0.06 ± 0.001 ^a^
Ultra-Trace and Toxic Elements	
Aluminium (Al)	13.0 ± 2.0
Boron (B)	5.4 ± 0.4
Barium (Ba)	4.0 ± 0.6
Strontium (Sr)	3.2 ± 0.4
Arsenic (As)	<0.05
Cadmium (Cd)	0.05 ± 0.005
Mercury (Hg)	<0.03
Lead (Pb)	0.02 ± 0.001
Bismuth (Bi)	-

^a^ The value of Ni expressed as µg/kg.

**Table 5:** Phenolic compounds and antioxidant activities of service tree fruits.

A. Total Phenolic Content, mg GAE/100 g	3574 ± 20
B. Total flavonoids content, mg QE/100 g	76.1 ± 1.0
Quercetin, mg/100 g	6.1 ± 0
Quercetin-3-*β*-glucoside	146 ± 12
Epicatechin	74 ± 2
Rutin	65 ± 2
Naringin	53 ± 7
Myricetin	40 ± 1
C. Total tannins, mg/100 g	122 ± 10
D. Phenolic acids, mg/100 g	
Neochlorogenic acid	908 ± 35
3,4-Dihydroxybenzoic acid	886 ± 15
Ellagic acid	26 ± 1
Ferulic acid	22 ± 1
E. Antioxidant activity	
DPPH assay, mM TE/g	11.3 ± 1.9
FRAP assay, mM TE/g	9.4 ± 0.3
ORAC assay, µmol TE/g	642.3 ± 31.9
HORAC assay, µmol GAE/g	67.8 ± 5.3

## Data Availability

Not applicable.

## Supplementary Materials

The following supporting information can be downloaded at: https://www.mdpi.com/article/10.3390/plants11141832/s1, Table S1: Amino acid score of service tree protein compared with the amino acids of standard and bovine milk protein, %; Table S2: Ratio of polyunsaturated (PUFA) and saturated (SFA) fatty acids, atherogenic, thrombogenic, and cholesterolemic indexes of the glyceride oil; Table S3: Sugar ratios, sweetness, total sweetness, sour and maturity indexes of service tree fruits. Figure S1: Calibration curves used for the quantification of flavonoid constituents of service tree fruits; Figure S2: Calibration curves used for the quantification of phenolic acids of service tree fruits.
