# Supplementary material for: Nutrient Constituents, Bioactive Phytochemicals, and Antioxidant Properties of Service Tree (Sorbus domestica L.) Fruits
Source: Plants (Basel). 2022 Jul 13;11(14):1832. doi: 10.3390/plants11141832 (PMC9323255; doi:10.3390/plants11141832)
Supplement: Supplementary file 1 [file plants-11-01832-s001.zip › plants-1763465-supplementary.pdf]

plants

Supplementary Material

Table S1. Amino acid score of service tree protein compared with the amino acids of standard and bovine milk protein, %.

Amino acids	Compared with indispensable amino acid requirements	Compared with the total protein of bovine milk
His	313	167
Val	149	85
Leu	93	53
Ile	130	63
Thr	140	67
Lys	102	55
Phe+Tyr	184	65
Met+Cys	186	141

Table S2. Ratio of polyunsaturated (PUFA) and saturated (SFA) fatty acids, atherogenic, thrombogenic, and cholesterolemic indexes of the glyceride oil.

Lipid indexes	Value
PUFA/SFA	2.0
Index of atherogenicity[a]	0.3
Index of thrombogenicity[b]	0.6
Cholesterolemic index[c]	3.8

[a]Index of atherogenicity = $(C_{12:0} + 4\times C_{14:0} + C_{16:0})/[\Sigma MUFA + \Sigma PUFA_{n\text{-}6 \text{ and } n\text{-}3}]$.
[b]Index of thrombogenicity = $(C_{14:0} + C_{16:0} + C_{18:0})/[0.5\times\Sigma MUFA + 0.5\times\Sigma PUFA_{n\text{-}6} + 3\times\Sigma\ PUFA_{n\text{-}3} + PUFA_{n\text{-}3}/PUFA_{n\text{-}6}]$.
[c]Cholesterolemic index = $\Sigma(C_{18:1} + C_{18:2} + C_{18:3n\text{-}3} + C_{18:3n\text{-}6} + C_{20:2} + C_{20:3} + C_{20:4} + C_{20:5} + C_{22:6})/\Sigma(C_{12:0} + C_{14:0} + C_{16:0})$.

Table S3. Sugar ratios, sweetness, total sweetness, sour and maturity indexes of service tree fruits.

Indexes and ratios	Value
Suc/Glc	0.12
Glc/Fru	0.72
Sweetness index	56.89
Total sweetness index	38.69
Sour index	1.47
Total sugars/Titrable acidity (Maturity index)	21.80

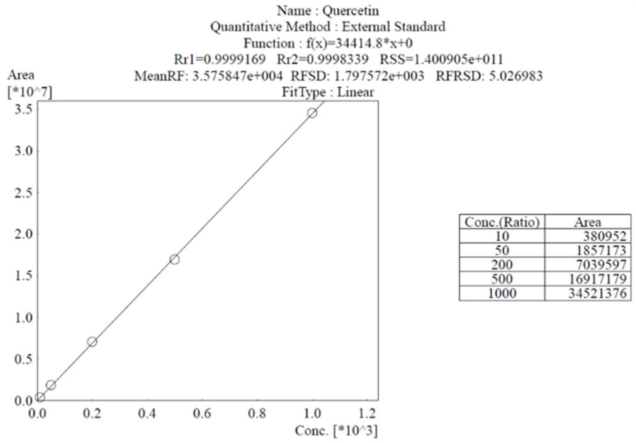

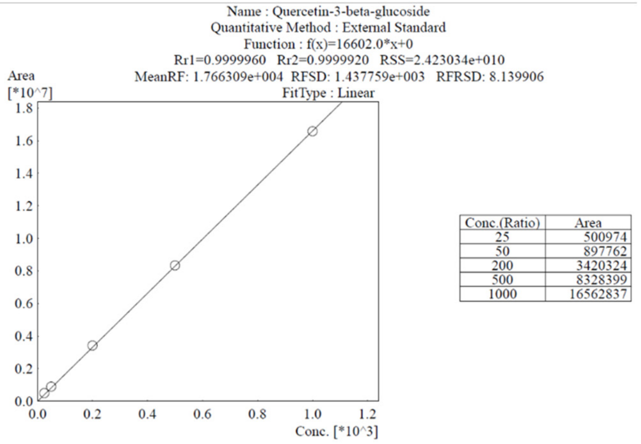

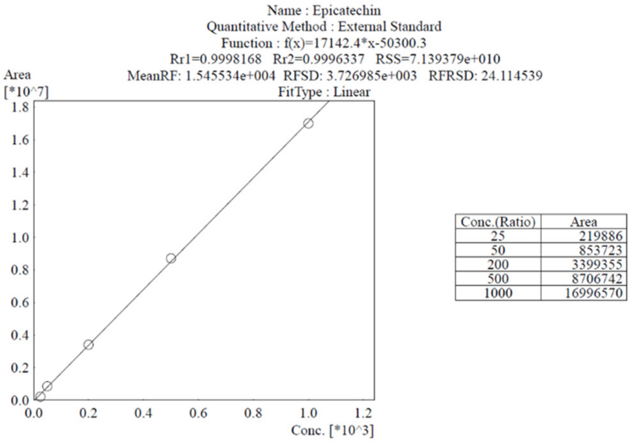

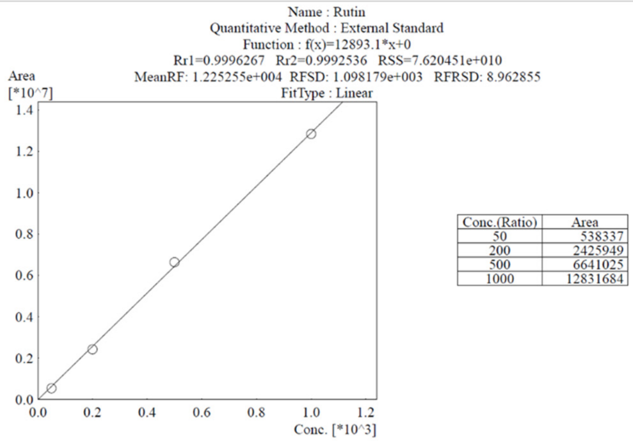

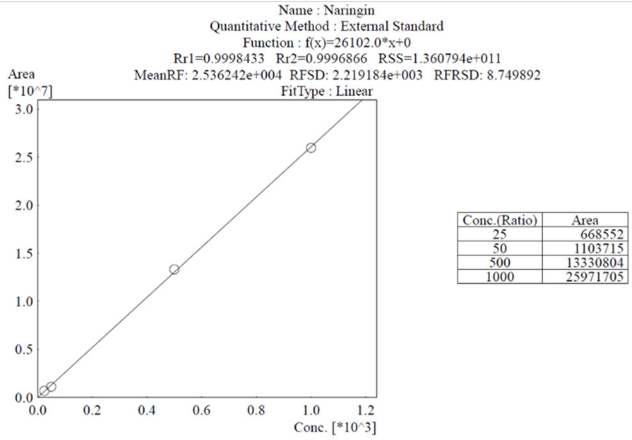

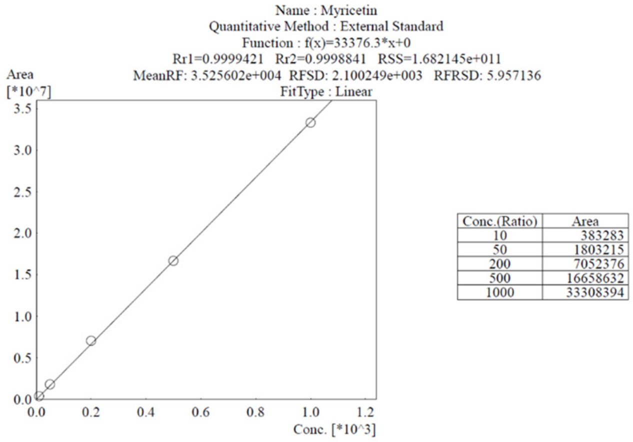

Figure S1. Calibration curves used for the quantification of flavonoid constituents of service tree fruits.

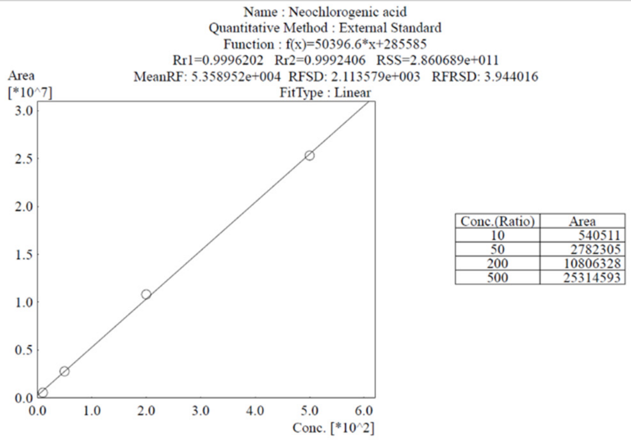

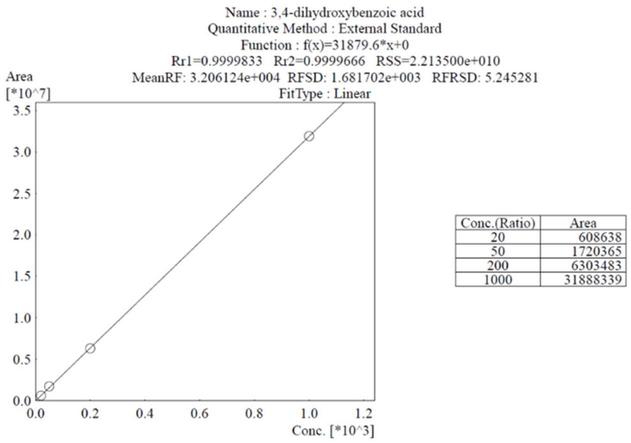

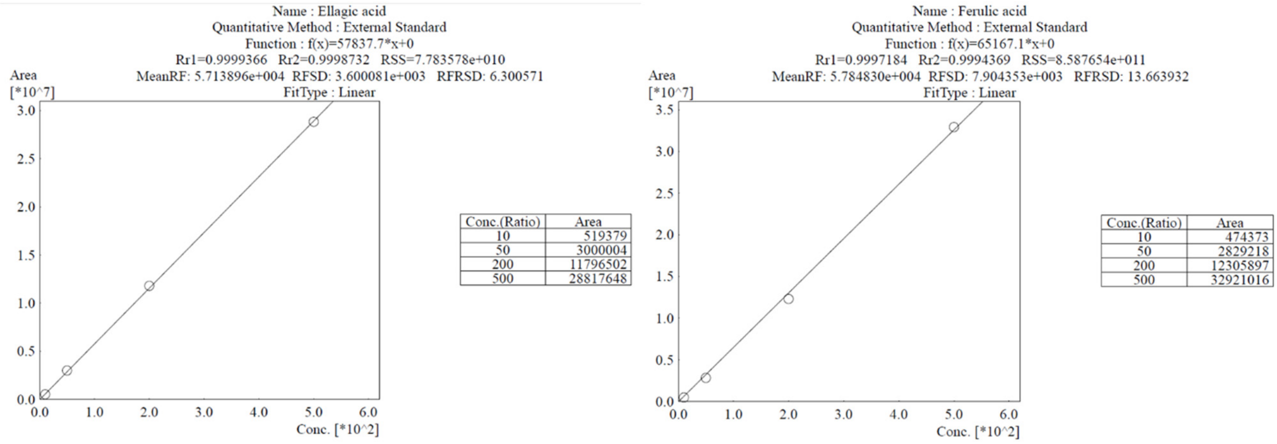

Figure S2. Calibration curves used for the quantification of phenolic acids of service tree fruits.
